# Pilot Survey of Knowledge, Attitudes and Perceptions of Hajj Deployed Health Care Workers on Antibiotics and Antibiotic Prescriptions for Upper Respiratory Tract Infections: Results from Two Hajj Seasons

**DOI:** 10.3390/tropicalmed5010018

**Published:** 2020-01-29

**Authors:** Hamid Bokhary, Osamah Barasheed, Moataz Abd El Ghany, Ameneh Khatami, Grant A. Hill-Cawthorne, Harunor Rasheed, Hajj Research Team

**Affiliations:** 1 School of Public Health, The University of Sydney, Sydney NSW 2006, Australia; grant.hill-cawthorne@sydney.edu.au; 2 University Medical Center, Umm Al-Qura University, Al Jamiah, Makkah, Makkah Region 24243, Saudi Arabia; 3 The Marie Bashir Institute for Infectious Diseases and Biosecurity, The University of Sydney, Westmead NSW 2145, Australiaharunor.rashid@health.nsw.gov.au (H.R.); 4 The Westmead Institute for Medical Research, Westmead NSW 2145, Australia; 5 The Executive Administration for Research and Innovation, King Abdullah Medical City, Al Mashair, Makkah, Makkah Region 24246, Saudi Arabia; 6 The Westmead Clinical School, Faculty of Medicine and Health, The University of Sydney, Westmead NSW 2145, Australia; 7 The Discipline of Child and Adolescent Health, The Faculty of Medicine and Health, The University of Sydney, Sydney NSW 2145, Australia; 8 National Centre for Immunisation Research and Surveillance (NCIRS), Kids Research Institute at The Children’s Hospital at Westmead, Westmead NSW 2145, Australia

**Keywords:** Saudi Arabia, Hajj, mass gathering, survey, health care workers, knowledge, attitudes, perceptions, antimicrobial resistance, antimicrobial stewardship, upper respiratory tract infection, guideline

## Abstract

Antimicrobial resistance (AMR) is a global public health issue. Upper respiratory tract infections (URTIs) are common illnesses during Hajj, for which antibiotics are often inappropriately prescribed. Hajj healthcare workers’ (HCW) knowledge, attitudes and perceptions (KAP) about AMR and antibiotic use for URTIs are not known. We conducted a survey among HCWs during Hajj to explore their KAP regarding antibiotic use for URTIs in pilgrims. Electronic or paper-based surveys were distributed to HCWs during the Hajj in 2016 and 2017. A total of 85 respondents aged 25 to 63 (median 40) years completed the surveys. Most participants were male (78.8%) and were physicians by profession (95.3%). Around 85% and 19% of respondents claimed to have heard about AMR and antimicrobial stewardship programs, respectively, among whom most had obtained their knowledge during their qualification. Implementation of URTI treatment guidelines was very low. In conclusion, HCWs at Hajj have significant knowledge gaps regarding AMR, often do not use standard clinical criteria to diagnose URTIs and display a tendency to prescribe antibiotics for URTIs.

## 1. Introduction

Antimicrobial resistance (AMR) is a growing global concern affecting human health. The World Health Organization (WHO) has addressed AMR on several levels: surveillance [1], action plans [2] and guidelines including antimicrobial uses other than for medical purposes [3]. With the increasing risk of AMR, it is predicted that it will be the main underlying reason for lives lost by the year 2050, with a predicted toll of 10 million people per year [4]. Tackling AMR requires a multi-disciplinary effort, including reducing unnecessary antimicrobial prescriptions by healthcare workers (HCWs) [5]. HCWs also play key roles in minimizing antimicrobial misuse through interventions such as promoting vaccination and infection control measures [5]. Therefore, assessing HCWs’ knowledge, attitudes and perceptions (KAP) on AMR is important to formulate an action plan to tackle drug resistance. This is crucially important in settings like travel, mass migration and during mass gatherings such as the Hajj pilgrimage in Makkah, Saudi Arabia.

Hajj is an annual religious mass gathering where over 2 million pilgrims assemble for around one week within a confined area measuring around 40 km^2^, where the risk of transmission of infectious diseases is amplified [6]. Hajj has already been associated with an increased risk of airborne, foodborne and zoonotic infections [7]. Recent studies have demonstrated that pilgrims are at high potential risk of acquiring and transmitting AMR enteric bacteria [8,9], including multidrug resistant *Acinetobacter* spp., carbapenemase-producing *Escherichia coli* [10] and extended-spectrum cephalosporin- and colistin-resistant non-typhoidal *Salmonella* [11]. Although there are studies documenting AMR acquisition during Hajj, there are little available data on the knowledge, attitudes and perceptions of HCWs regarding antimicrobial use for the treatment of upper respiratory tract infections (URTIs) for pilgrims.

URTI is the most common medical complication among Hajj pilgrims [12,13,14,15], and studies showed that the majority of URTIs are treated with antibacterial agents (henceforth referred to as antibiotics without specification) [16], even though up to 95% at onset of symptoms, during Hajj, are known to be viral [17]. A recent study revealed that around 93% of pilgrims develop respiratory symptoms, of which 78% of tested samples were positive for at least one pathogen [18]. The most commonly found bacterial causes for respiratory infections were *Haemophilus influenzae* and *Staphylococcus aureus*, and viral causes were human rhinoviruses [19,20,21]. Since 1978, a law has been passed in Saudi Arabia prohibiting pharmacists from dispensing any drug, including antibiotics, without a prescription issued from a licensed physician, unless excluded by the Ministery of Health as “over-the-counter” medications [22]. In Saudi Arabia, a physician is recognised as anyone with a university medical degree, that is approved by the Saudi Commission For Health Specialties to practice medicine in Saudi Arabia [22]. However, enforcing this legislation is still a struggle [23]. In 2011, around 40% of requests to pharmacists in Saudi Arabia were for antibiotics without a physician’s prescription; 98% of these requests were obliged [24]. Moreover, in Hajj contexts, it has previously been noted that 78% of antibiotics used by Australian Hajj pilgrims were dispensed without a physician’s prescription [25]. These proportions are expected to improve further with the Saudi government enforcement of the drug dispencing law. However, to our knowledge, there are no antimicrobial stewardship programs (ASPs) that are specific to Hajj; moreover, with the exception of the 2009 influenza A(H1N1) pandemic, there are no readily available local guidelines for treating URTIs in the Hajj setting.

In this survey, we attempted to assess the KAP of Hajj deployed HCWs regarding antibiotics, AMR, URTIs and their understanding of evidence-based medicine (EBM) practices that are specific to treating URTIs; such as the use of Centor criteria and the National Institute for Health and Care Excellence clinical guideline 69 (NICE-CG69) [26,27]. The latter of which, as of September 2019, is under review and is expected to be updated [28].

## 2. Methods

### 2.1. Setting and Study Questionnaire

The study was conducted over two Hajj seasons (2016 and 2017). On both occasions, the Hajj Research Team were recruited via online portals and networking. The team members were senior university students or graduates who were residents of Makkah city at the time of Hajj. Transportation was provided to designated catchment areas for conducting the survey, such as Mina, Aziziya and Holy Mosque areas in Greater Makkah. The research team visited HCWs, primarily physicians, in their place of work during the peak period of the Hajj ritual week and invited them to take part in the survey after providing relevant study information. The anonymous survey was initially conducted online in 2016; however, due to a low response rate, a paper-based questionnaire was used in 2017. Both questionnaires are provided in the Supplementary Materials (Document S1). Completion of the survey was taken as implied consent to participate in the research, and the study protocol and related documents were approved by the King Abdullah Medical City Institutional Review Board (IRB number: 16-293).

### 2.2. Participants

For the 2016 Hajj season, recruitment of HCWs was done by 39 research team members (18 female). Following verbal consent of HCWs, an online link to the survey was sent to potential participants. Links and email invitations were generated and sent through the RedCap^®^ (Vanderbilt University, Nashville, TN, USA) system. Responses could only be submitted when complete; they were stored as incomplete if not submitted or not answered.

For the 2017 Hajj season, recruitment of HCWs was done by five research team members (only one female). A paper-based survey was distributed among HCWs after they had consented to participate. The responses were subsequently entered by the first author (HB) into RedCap^®^.

Inclusion criteria were that the respondent should be an HCW, work during Hajj, work in a facility that may serve pilgrims and have the authority to prescribe or dispense antibiotics.

### 2.3. Data Analysis:

Raw data were extracted from RedCap^®^ and cleaned through Excel 2016 (Microsoft Office 2016, Microsoft Corporation, Redmond, Washington, DC, USA). The data were exported to SPSS^®^22 (SPSS^®^ Inc., IBM^®^ Corporation, Armonk, New York, NY, USA.) for analysis. Missing data were excluded from the analysis for each field, with the denominator representing all valid responses for each question.

## 3. Results

**Figure 1:**
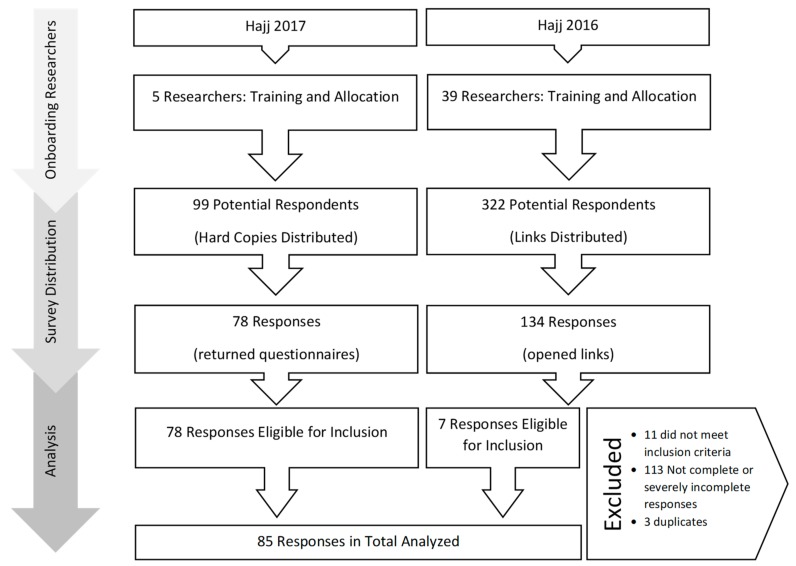
Flowchart for recruitment and analyzed responses.

Figure 1 outlines the recruitment of HCWs and response rates to the questionnaires in each year of the study. Sixty-seven out of the included 85 respondents (78.8%) were male. Other demographic and Hajj deployment related information are provided in Table 1.

Most (73/80, 91.3%) HCWs felt that their working conditions were “crowded” with respect to patient visits during Hajj. Among respondents stating they saw more than 25 patients/day, at least one response was up to 200 patients/day. Interestingly, regardless of HCWs’ perception of crowding in the workplace, 68.6% (48/70) of HCWs stated that their decision to prescribe antibiotics was not affected by the patient load on their services. Only 20.0% (14/70) would prescribe fewer antibiotics during Hajj than in non-Hajj contexts.

### 3.2. KAP Regarding Antibiotics and AMR

The proportion of Hajj deployed HCWs who were aware of reports of resistance to antibiotics ranged from 84.7% (61/72) for penicillin to 22.0% (13/59) for colistin. Interestingly, four HCWs were not aware that colistin (two HCWs), isoniazid (three HCWs), pyrazinamide (two HCWs) and ethambutol (one HCW) were antibiotics. An overview of HCWs’ KAP regarding regulatory procedures for, and use of, antibiotics are found in Table 2.

### 3.3. KAP Regarding URTIs and Antibiotic Prescription for URTIs

The majority (71.8%, 61/74) of Hajj deployed HCWs believed there should be guidelines for prescribing antibiotics for URTIs during Hajj. Specific reasons included the need for Hajj health services to be evidence-based (51/61, 83.6%), to save time (40/61, 65.6%) and for greater standardization of health services (33/61, 54.1%). A summary of the KAP of HCWs deployed during Hajj regarding URTIs and related management is provided in Table 3.

Only 3.6% (1/28) of those who knew of NICE-CG69 practiced it correctly; a surprisingly lower proportion than among those who denied knowledge of its existence (20.0%, 3/15). 32/72 (44.4%) Hajj deployed HCWs would, as per the guideline, advise their patients that antibiotics were not required when not prescribing antibiotics for URTIs, compared with 20.8% (15/72) and 6.9% (5/72) of those providing immediate or delayed prescriptions, respectively. Only 5.0% (1/20) of those who knew of the Centor criteria provided responses to demonstrate correct use of such criteria, whereas 6.9% (2/29) of HCWs who claimed not to have heard of the Centor criteria were practicing the guideline correctly.

## 4. Discussion

This survey demonstrates a substantial gap in knowledge about antibiotics, AMR and antibiotic treatment protocols for URTIs among HCWs deployed during Hajj. The key results are the knowledge gaps identified with respect to bacterial resistance to specific antibiotics, ASP and pathogens treated by antibiotics. HCWs should know that antibiotics do not treat viruses; however, Hajj deployed HCWs’ understanding that antibiotics do not treat viruses (36%) is only slightly higher than that of the Saudi general public (24%) [29]. This may not reflect a true lack of knowledge about antibiotics but could be due to lack of clarity about the questions since several conflicting results were identified that suggest some HCWs were confused with the use of English terms such as ‘antimicrobial’ and ‘antibacterial’. As such, we would recommend future studies use questionnaires with Arabic translations when addressing HCWs in Hajj.

Although HCWs’ knowledge regarding AMR is not lower than the perceived global clinician HCW average (90% vs. 69%) [30], most HCWs surveyed did not know about ASP, which are relatively new and were not prominent in the medical literature at the time of most respondents’ graduation [31]. This highlights the importance of continuing medical education (CME), which is mandatory for physicians working under most jurisdictions, and suggests greater emphasis on education on AMR and ASP is required for HCWs in such settings [32]. Nevertheless, all of the valid responses from Hajj deployed HCWs with respect to their attitudes towards various methods of restricting antibiotics were positive.

Awareness among Hajj deployed HCWs regarding the existence of guidelines for prescription of antibiotics for URTIs is low, and their reported compliance with such guidelines is even lower. This is a commonly encountered problem in many settings. For instance, a study conducted in Boston, USA found that even though several guidelines were available on prescribing antibiotics for URTIs, up to 66% of physicians failed to adhere to those guidelines [33]. Among HCW surveyed in this study, there was a tendency to prescribe amoxicillin and/or azithromycin as the first choice of antibiotics (96%). Data derived from the World Health Organization’s Eastern Mediterranean Region also demonstrated high consumption of these two drugs, although the report does not show if there was any association between the consumption of amoxicillin and/or azithromycin and the treatment of URTI [34]. While our survey did not specifically ask about treatment of URTIs in the context of Hajj, the responses received suggest that the same practices would occur during the Hajj season. The limited available literature on this topic suggests low appropriateness of antibiotic prescriptions during Hajj. Only around 40% of French pilgrims who were prescribed antibiotics in one report had an appropriate indication according to French recommendations [35]. Similarly, another study found 98.6% of patients attending an ear, nose and throat clinic during Hajj were prescribed antibiotics, even though 44.6% were diagnosed with viral infections [16]. We suggest that recommendations for treating URTIs during Hajj should be devised and their implementation studied by local authorities, in response to requests from HCWs to have such guidelines readily available.

Some of the findings of this survey may be due to a lack of incorporation of EBM principles in the undergraduate medical curriculum. A study of medical students in Saudi Arabia found that 70% did not attend EBM workshops and only 24.4% would follow such evidence [36]. Moreover, negligible EBM training in CME programs and limited access to EBM resources might have further widened this knowledge gap [37]. Factors related to high patient loads may also affect implementation of knowledge into practice. Most of the Hajj deployed HCWs surveyed worked for 84 h a week, mostly while working 12 h shifts, during which most saw at least 182 patients per week. In comparison, family physicians in the USA work for about 51 h a week, for a working day of about 8.5 h, and see an average of 99 patients per week [38].

Limitations of this survey include missing information; potential language barriers and misunderstanding of terminology; a small sample size that is skewed to male respondents; limitations of the survey questions that did not fully address the effect of CME on HCW’s KAP regarding AMR, use of antiviral agents or specific differences between Hajj and non-Hajj settings. Moreover, there were reports from participants that the survey was too long for Hajj contexts. As highlighted above, future research in this area should incorporate a larger and more diverse participant population, with surveys conducted in Arabic in addition to, or instead of, English. In addition, it would be useful to reconduct similar surveys over several years to assess for improvements in Hajj deployed HCWs’ KAP regarding AMR, antibiotics and URTI treatment. Furthermore, additional insights regarding justifications for antibiotic use may be gained through semi-structured focus-group interviews involving physicians deployed during Hajj.

## 5. Conclusions

Hajj deployed HCW have low awareness about AMR, ASP and EBM principles. Some lack precise knowledge about antibiotics and their use. Most HCWs at Hajj do not use standard criteria to diagnose URTIs and they work long hours with high patient loads. Specific education on AMR, ASP and EBM implementation should start early in medical training, and continue through practicing years. Further studies are needed to address potential benefits that may be gained from Hajj-specific antibiotic guidelines and antimicrobial stewardship programs.

## Figures and Tables

**Table 1:** Demographics and responses to Hajj deployment related questions.

Query	Valid Response	Count; n (%)	
**Gender**	Male:Female	4.2:1	
Age	Years; Median (Range)	40	(25–63)
Nationality	Saudi Arabia	27	(33.7)
	Egypt	24	(30.0)
	Sudan	18	(22.5)
	Pakistan	9	(11.3)
	Other ^1^	2	(2.5)
	Missing	5	
Occupation	Physician	81	(95.3)
	Pharmacist	3	(3.6)
	Nurse	1	(1.2)
Qualification Level	Bachelor degree (Physicians)	33	(39.2)
	Bachelor degree (Other)	3	(3.6)
	Specialist	36	(42.9)
	Consultant	11	(13.1)
	Diploma ^2^	1	(1.2)
	Missing	1	
Stationing during Hajj	Hospitals	69	(94.5)
	Primary Healthcare Centers	3	(4.1)
	Community Pharmacy	1	(1.4)
	Unspecified or missing	12	
Number of previous Hajj seasons of deployment	First time	29	(35.4)
	1–3 times	22	(26.8)
	4–9 times	23	(28.0)
	≥10 times	8	(9.8)
	Missing	3	
Hajj working days ^3^	≤15 days	70	(89.7)
	≥20 days	8	(10.3)
	Unspecified or missing	7	
Hajj working hours (daily shift)	8 h shift	17	(22.4)
	12 h shift	59	(77.6)
	Unspecified or missing	9	
Number of patients seen daily at Hajj	≤25 patients/day	22	(29.3)
	≥26 patients/day	53	(70.7)
	Unspecified or missing	10	

^1^ India (1) and Syria (1); ^2^ Pharmacist; ^3^ There were no responses documented for 16–19 days.

**Table 2:** Knowledge and attitude of Hajj deployed healthcare workers regarding regulations and uses for antibiotics.

Query	Valid Responses	n (%) Out of (N); Missing		
Did you hear about antimicrobial resistance?	Yes—from any source	72	(90.0)	80; 5
	Yes—from academic studies	56	(80.0)	70; 2
	Maybe	7	(8.8)	80; 5
	No	1	(1.2)	80; 5
Did you hear about antimicrobial stewardship programs?	Yes—from any source	16	(20.0)	80; 5
	Yes—from academic studies (qualification at any time)	10	(66.7)	15; 1
	Academic qualification—Post-1996	9	(90.0)	10; 0
	Maybe	18	(22.5)	80; 5
	No	46	(57.5)	80; 5
Physician’s recommendation should be required for dispensing antibiotics		76	(98.7)	77; 8
Antibiotics should only be dispensed with a prescription		70	(93.3)	75; 10
There should be compliance visitations to pharmacies by the governing body		65	(87.8)	74; 11
There should be evidence-based criteria for antibiotic prescriptions		72	(93.5)	77; 8
Do antibiotics treat bacterial infections in Hajj?	Yes—during Hajj	72	(93.5)	77; 8
	Yes—also in non-Hajj contexts as a potential choice of treatment	69	(100.0)	69; 3
	Yes—in both contexts but as the main choice of treatment	39	(58.2)	67; 2
	Maybe	4	(5.2)	77; 8
	No	1	(1.3)	77; 8
Is there evidence that antibiotics treat bacterial infections in Hajj?	No evidence supporting antibiotic used for treatment	5	(6.7)	75; 10
	No evidence to refute antibiotics as treatment	22	(29.3)	
Do antibiotics treat viral infections in Hajj?	Yes—during Hajj	10	(12.5)	80; 5
	Yes—also in non-Hajj contexts as a potential choice of treatment	3	(50.0)	6; 4
	Yes—in both contexts but as the main choice of treatment	2	(66.7)	3; 0
	Maybe	13	(16.3)	80; 5
	No—not during Hajj	57	(71.2)	80; 5
	No—does not treat also in non-Hajj contexts	32	(74.4)	43; 14
	No—in both contexts, but would treat for any reason if “warranted”	8	(32.0)	25; 7
	Warranted—Secondary bacterial infections	5	(71.4)	7; 1
Is there evidence that antibiotics treat viral infections in Hajj?	No evidence supporting antibiotics used for treatment	31	(40.8)	76; 4
	No evidence to refute antibiotics as treatment	11	(14.5)	

**Table 3:** Knowledge and perceptions of Hajj deployed healthcare workers regarding upper respiratory tract infections (URTIs) and related treatment information.

Query	Valid Response	Count; n	(%)
Perception of the proportion of patients presenting with tonsillitis	More in Hajj context	15	(24.6)
	The same in Hajj and non-Hajj contexts	29	(47.5)
	More in non-Hajj context	21	(34.4)
	Missing	24	
Perception of the proportion of patients presenting with common cold	More in Hajj context	9	(13.4)
	The same in Hajj and non-Hajj contexts	33	(49.3)
	More in non-Hajj context	25	(37.3)
	Missing	18	
Perception of the proportion of patients presenting with sore throat	More in Hajj context	6	(9.1)
	The same in Hajj and non-Hajj contexts	40	(60.6)
	More in non-Hajj context	20	(30.3)
	Missing	19	
First choice of treatment for URTI	amoxicillin alone	26	(34.7)
	azithromycin alone	9	(12.0)
	amoxicillin with antibiotics other than azithromycin	22	(29.3)
	azithromycin with antibiotics other than amoxicillin	4	(5.3)
	antibiotics including amoxicillin and azithromycin	11	(14.7)
	antibiotics other than amoxicillin and azithromycin	3	(4.0)
	Missing	10	
Knowledge of existence of NICE-CG69 ^1^	Yes	29	(40.8)
	No	23	(32.4)
	Not sure	19	(26.8)
	Missing	14	
Knowledge of existence of Centor criteria	Yes	22	(31.9)
	No	33	(47.8)
	Not sure	14	(20.3)
	Missing	16	

^1^ National Institute for Health and Care Excellence clinical guideline 69.

## Supplementary Materials

The following are available online at: https://www.mdpi.com/2414-6366/5/1/18/s1, Document S1: Survey questionnaire.
