# Supplementary material for: Pilot Survey of Knowledge, Attitudes and Perceptions of Hajj Deployed Health Care Workers on Antibiotics and Antibiotic Prescriptions for Upper Respiratory Tract Infections: Results from Two Hajj Seasons
Source: Trop Med Infect Dis. 2020 Jan 29;5(1):18. doi: 10.3390/tropicalmed5010018 (PMC7157631; doi:10.3390/tropicalmed5010018)
Supplement: Supplementary file 1 [file tropicalmed-05-00018-s001.pdf]

Antimicrobial Prescription Knowledge, Attitude and Practices of Physicians deployed for Hajj during 2017G (1438H) Hajj season

Dear Participant,

Thank you for the time that you'll give to fill out this form. This study helps in understanding the nature of antimicrobial prescription during the Hajj season. You are invited to participate in this survey if you are a Heath Care Worker deployed during Hajj. This survey is part of studies conducted by researchers from The University of Sydney (Sydney, Australia) in cooperation with their counterparts from King Abdullah Medical City (Makkah, Saudi Arabia).

King Abdullah Medical City has ethically approved this survey. Further information regarding the approval is on their website. Ethics approval number 16-293.

This study is not in any way related to your work, job requirements or assessment. The provided information will only be used for the sole purpose of the studies and will not be distributed to other parties. You can ask for the information sheet, if not provided already, for your future reference from the primary investigator (e-mail found below, please quote "2017 KAP Participant Information Sheet" in the subject field).

Thank you for your contribution,

Hamid Bokhary, Primary investigator, on behalf of the Hajj Research Team.
E-mail: hamid.bokhary@sydney.edu.au

1. Kindly provide the name or reference number of the researcher that approached you for this survey this time. (If researcher is filling this please write your number)

2. Who is filling this survey?
   - o I am filling this on my own as a participant.
   - o I was interviewed through a researcher.

3. Most of this survey's questions don't require writing the answers.
   Please just tick (✓) the appropriate circle with the suitable choice when presented.
   - o I understand and will bear it in mind when answering.

4. You will find the term "Health Care Worker" repeated in this survey.
   Health Care Worker(s) will be defined as:
   A person (or people) who usually work(s) or train(s) in a health care facility such as a hospital, walk-in clinic, dispensary, pathology laboratory, public health office and emergency care services. Thus, would include: trainee doctors, nurses, allied HCWs, pharmacists, community pharmacists, laboratory technicians and ambulance personnel.
   A trainee will be defined as:
   Someone who is still undergoing training such as medical, nursing or pharmacology student undergraduates; or a postgraduate (e.g.: physician or pharmacist) in vocational specialty training.
   - o I understand and will bear it in mind when answering.

5. You will find the term "client" repeated in this survey.
   Health care beneficiaries or receivers are not always patients, like those who go for routine check-ups.
   In this survey a client is defined as:
   The end receiver of any health care related service; treatments, check-ups, lab results, medical tests, rehabilitation, medical care, consultation and home visits are considered as such. Patients and those who come asking for medication are considered clients in this survey.
   Hence a "Hajj client" is a "client" but presents himself as a pilgrim during Hajj season.
   - o I understand and will bear it in mind when answering.

6. Please bear in mind that this is NOT a test of your knowledge as an individual, but its intent is to seek the baseline knowledge of on-the-ground Health care workers in Hajj.
   - o I understand and will bear it in mind when answering.

7. Declaration:
   - o I am a Health Care Worker and I am allowed to practice my profession in Hajj. (pharmacist, nurse, physician, ...etc.)
   - o I am deployed during this Hajj season (1438H/2017G) to work as a Health Care Worker.

8. Is this your first time filling this survey during this season of Hajj (1437H/2016G)?
   - o Yes (go to Question number 11)
   - o No

9. If you answered Question 8 with "No", please mention how many times did you fill this survey (not including this one):

10. If you answered Question 8 with "No", why are you filling it again this time?

11. What is your gender?
    - o Male
    - o Female

12. What is your year of birth? (Gregorian calendar)

13. What is your country of birth?

14. What is your nationality?

15. What is your current qualification?
    - Still a student, including last year medical students (Internes/House Officers)
    - Diploma graduate
    - High Diploma or Intermediate University Degree graduate
    - Bachelors graduate (including MBBS)
    - Postgraduate Diploma in a subspecialty. (Like Family Medicine Diploma)
    - Masters graduate
    - PhD graduate
    - Board qualified
    - Medical/Health Specialist
    - Medical/Health Consultant
    - Other, please specify:

16. What is your qualification background / basis of your qualifications?
    - Physician (Medical Doctor)
    - Pharmacist
    - Nursing
    - Lab scientist
    - Administrative (Management)
    - Allied Health
    - Other, please specify:

17. Year of obtaining qualification: (Gregorian Calendar)

18. Country granting the current qualification:

19. Are you working in the scope of your qualification?
    - Yes
    - Yes, but in administrative capacity (e.g.: Physician working as head of department)
    - No, in a different scope. (e.g.: Physician working in management)
    - Other, please specify:

20. Where are you stationed this year (2017G/1438H) during Hajj?
    (What is the name of the place of your work during Hajj)

21. How many Years have you worked as a Health Care Worker in general?
    - still a Student
    - less than a year
    - 1-3 years
    - 4-9 years
    - more than 10 years

22. How many Hajj seasons have you worked as a Hajj Health Care Worker?
    - This is my first season
    - 1-3 seasons
    - 4-9 seasons
    - more than 10 seasons

23. What is the average number of days per year you work in Hajj as a Hajj Health Care Worker. (if this is your fist time then please specify number of days you are planning to work for)
    - 15 days
    - 13 days
    - Other, please specify:

24. What is the average number of daily hours you put in Hajj? (as a Health Care Worker in Hajj)
    - 12 hours
    - 8 hours
    - 6 hours
    - Other, please specify:

25. How many "Hajj clients" do you see in Hajj (as a Health Care Worker in Hajj) in average?
    - less than 5 Hajj clients / day
    - 5-15 Hajj clients / day
    - 15-25 Hajj clients / day
    - more than 25 Hajj clients / day

26. Have you ever heard of Antimicrobial Resistance?
    - Never heard of it. (go to Question number 28)
    - Maybe or minimal information
    - Yes, I know it well
    - Other, please specify:

27. If you answered Question 27 with "Yes" or "Maybe", where did you hear it from? (you may choose more than one)
    - Newspaper
    - Web
    - My degree/specialty course/training
    - Extracurricular Academic Reading. (Including Academic Paper/Newsletter)
    - Other, please specify:

28. Have you ever heard of Antimicrobial Stewardship Programs?
    - Never heard of it. (go to Question number 30)
    - Maybe or minimal information
    - Yes, I know it well
    - Other

29. If you answered Question 29 with "Yes" or "Maybe", where did you hear it from? (you may choose more than one)
    - o Newspaper
    - o Web
    - o My degree/specialty course/training
    - o Extracurricular Academic Reading. (Including Academic Paper/Newsletter)
    - o Other, please specify:

30. What organisms do you think antimicrobials help treat (in general, not specific to Hajj)? (Please choose one per organism)

Organism	Yes	No	Maybe	I don't Know
Viruses	O	O	O	O
Bacteria	O	O	O	O
Fungi	O	O	O	O
Parasites	O	O	O	O
Insects	O	O	O	O

31. Do you think antimicrobials should be available to the public without restrictions?
    - o Antimicrobial availability should be restricted to the public. (go to Question number 33)
    - o NO restrictions should apply to the availability of antimicrobials to the public

32. If you answered "NO restrictions should apply to the availability of antimicrobials to the public" previously, please mention below why:

33. If you answered for restrictions previously, what restrictions should be applied for dispensing antimicrobials?

Restrictions	Yes	No	Maybe
Antimicrobials need a doctor's opinion for consideration	O	O	O
Dispensaries should only give antimicrobials if presented with a prescription	O	O	O
Only one type of antimicrobials should be dispensed to a recipient	O	O	O
Manufacturing of antimicrobials should be regulated and supervised	O	O	O
Regular and unplanned inspections to Pharmacies, to assess policy adherence.	O	O	O
Doctors should only prescribe antimicrobials if an evidence based criterion was achieved	O	O	O

34. Please share below any other restrictions: (optional)

35. Sometimes antimicrobials would "not to work" anymore against the same illness, what would the reason for that be?

Reason	Yes	No	Maybe	I don't Know
The human body builds up immunity/resistance against the antimicrobial	○	○	○	○
The organisms build up immunity/resistance against the antimicrobial	○	○	○	○
The antimicrobial efficacy decreases against the illness over time	○	○	○	○

36. Please share below any other reasons: (optional)

37. what best describes your authority in regards to antimicrobial prescription in Saudi Arabia or Hajj?
   - I CANNOT prescribe antimicrobials in Saudi Arabia or Hajj. (Please return this survey to the research team)
   - I CAN prescribe antimicrobials in Saudi Arabia or Hajj

38. Are you a physician Health Care Worker?
   - Yes
   - No, please specify why do you have the authority to prescribe without being a physician?

39. What is your specialty?
   - Intern, Year 7 Medical School
   - General Practitioner (GP), Medical school graduate who's not enrolled in a speciality
   - Family Medicine
   - ENT (Ear, Nose and Throat)
   - Public Health/Community Medicine
   - Emergency Medicine
   - Paediatrics
   - Surgery
   - Internal Medicine
   - Other, please specify:

40. Do antimicrobials treat Viruses (in Hajj setting)?
   - Yes (go to Question number 42)
   - No
   - Maybe
   - I don't know

41. If you answered Question number 40 with "No", "I don't know" or "Maybe" would you prescribe anyway?
    - No
    - Yes, all the time
    - Yes, in specific situations like:

42. Regardless of your answer above, why would you prescribe (or not) antimicrobials? (You may choose more than one)
    - It is the main choice of treatment
    - To stop further deterioration of the client's clinical situation
    - There is no evidence supporting GIVING antimicrobials during Hajj against Viruses
    - There is no evidence supporting NOT GIVING antimicrobials during Hajj against Viruses
    - Other, please specify:

43. Do antimicrobials treat Bacteria (in Hajj setting)?
    - Yes (go to Question number 45)
    - No
    - Maybe
    - I don't know

44. If you answered Question 43 with "No", "I don't know" or "Maybe" would you prescribe anyway?
    - No
    - Yes, all the time
    - Yes, in specific situations like:

45. Regardless of your answer above, why would you prescribe (or not) antimicrobials? (You may choose more than one)
    - It is the main choice of treatment
    - To stop further deterioration of the client's clinical situation
    - There is no evidence supporting GIVING antimicrobials during Hajj against Bacteria
    - There is no evidence supporting NOT GIVING antimicrobials during Hajj against Bacteria
    - Other, please specify:

46. Are you crowded with clients during Hajj?
    - No, it is like any other given day
    - Yes, but it is bearable and manageable
    - Yes, in an intolerable amount. (Too much clients)
    - Other, please specify:

47. Regardless of your answer, does your situation above affect your decision on prescribing antimicrobials?
    - No, it does not
    - Yes, I tend to prescribe MORE antimicrobials during Hajj
    - Yes, I tend to prescribe LESS antimicrobials during Hajj
    - Other, please specify:

48. Have you heard reports of Antimicrobial Resistance for the antimicrobials below?

Antimicrobial	Yes	No	Maybe	Not an antimicrobial	Don't Know
Penicillin	O	O	O	O	O
Metronidazole	O	O	O	O	O
Amoxycillin	O	O	O	O	O
Amoxycillin with clavulanic acid	O	O	O	O	O
Ciprofloxacin	O	O	O	O	O
Rifampicin	O	O	O	O	O
Tetracyclines	O	O	O	O	O
Doxycycline	O	O	O	O	O
Ethambutol	O	O	O	O	O
Isoniazid	O	O	O	O	O
Pyrazinamide	O	O	O	O	O
Streptomycin	O	O	O	O	O
Colistin	O	O	O	O	O
Trimethoprim with Sulfamethoxazole	O	O	O	O	O
Erythromycin	O	O	O	O	O
Cephalosporin	O	O	O	O	O
Gentamicin	O	O	O	O	O

49. Do you treat clients for Upper Respiratory Tract Infections?
    - No, even though it is within the scope of my speciality
    - No, it is NOT within the scope of my speciality
    - Yes, it is within the scope of my speciality
    - Yes, but it is NOT within the scope of my speciality

50. Do you treat clients for Gastrointestinal Tract infections?
    - No, even though it is within the scope of my speciality
    - No, it is NOT within the scope of my speciality
    - Yes, it is within the scope of my speciality
    - Yes, but it is NOT within the scope of my speciality

51. If you were overwhelmed with clients during Hajj, would you prescribe antimicrobials to ALL your Hajj clients when treating them from Upper Respiratory Tract Infections?
    - Yes (go to Question number 52)
    - No (go to Question number 53)

52. If you have answered Question 51 with “Yes”, why would you?
    (You may choose more than one)
    - I DO NOT have time to assess each client's detailed case, because Hajj settings are usually crowded
    - Other, please specify:

    (go to Question number 54)

53. If you have answered Question 51 with “NO”, why would you?
    (You may choose more than one)
    - I have to assess each client as a unique case, as long as it takes to do so, bearing in mind Hajj settings are usually crowded
    - Other, please specify:

54. If you were overwhelmed with clients during Hajj, would you prescribe antimicrobials to ALL your Hajj clients when treating them from Gastrointestinal Tract Infections?
    - Yes (go to Question number 55)
    - No (go to Question number 56)

55. If you have answered Question 54 with “Yes”, why would you?
    (You may choose more than one)
    - I DO NOT have time to assess each client's detailed case, because Hajj settings are usually crowded
    - Other, please specify:

    (go to Question number 57)

56. If you have answered Question 54 with “No”, why would you?
    (You may choose more than one)
    - I have to assess each client as a unique case, as long as it takes to do so, bearing in mind Hajj settings are usually crowded
    - Other, please specify:

57. After prescribing an antimicrobial for Upper Respiratory Tract Infections to a Hajj client, have you ever considered advising them to delay taking it (not starting regimen immediately)?
    - Yes, always
    - Yes, for some clients
    - No, never (go to Question number 59)

58. Please elaborate: What is the percentage of clients with Upper (%) Respiratory Tract Infections you advise to delay taking antimicrobials that you already prescribed for them?

59. Regardless of your answer, why? (You may choose more than one)
    - If I wanted to delay treatment, I would not have prescribed antimicrobials in the first place
    - I want to give the client's natural immunity a chance to fight the illness
    - Other, please specify:

60. From the following practices, what would you offer your clients during Hajj? (You can choose more than one)

	When refusing to prescribe antimicrobials	When delaying Prescribing antimicrobials	When giving Antimicrobials immediately	Never do it
Reassurance that antimicrobials are not needed immediately because they are likely to make little difference to symptoms and may have side effects, for example, diarrhoea, vomiting and rash	○	○	○	○
Advice to re-consult if there is a significant worsening or prolongation of symptoms	○	○	○	○
Advice about the usual natural history of the illness, including the average total length of the illness (before and after seeing you)	○	○	○	○
Advice about managing symptoms, including fever (particularly analgesics and antipyretics)	○	○	○	○

61. Do you like to share any other practices in regards to prescribing antimicrobials in Hajj? (Optional)

62. Would you prescribe antimicrobials to the following clinical scenarios during Hajj?

	Yes alone	Yes with 1 other scenario	Yes with 2 other scenarios	Yes with ≥ 3 scenarios	Yes if absent	Never	Don't know
Old / geriatric clients (>55 years old)	○	○	○	○	○	○	○
Young clients (2 to < 15 years old)	○	○	○	○	○	○	○
Very Young clients (< 2 years old)	○	○	○	○	○	○	○
Client is systematically unwell	○	○	○	○	○	○	○
If clinical assessment suggests serious illness/complications	○	○	○	○	○	○	○
If client has a pre-existing comorbidity (heart, lung, renal, liver ...etc.)	○	○	○	○	○	○	○
If client has a pre-existing chronic medical illness (diabetes...etc.)	○	○	○	○	○	○	○
History of hospitalization in previous year	○	○	○	○	○	○	○
Bilateral acute otitis media in children younger than 2 years	○	○	○	○	○	○	○
Acute otitis media in children with otorrhoea	○	○	○	○	○	○	○
Three or more Centor score/criteria are present	○	○	○	○	○	○	○
Fever (≥ 38.0 °c)	○	○	○	○	○	○	○
The presence of exudative tonsillitis (Pus on tonsils)	○	○	○	○	○	○	○
The presence of lymphadenopathy or lymph node enlargement in relevant areas	○	○	○	○	○	○	○
Cough	○	○	○	○	○	○	○
Use of oral glucocorticoids	○	○	○	○	○	○	○

63. Would you like to share any other scenarios that you would prescribe antimicrobials for? if so, please mention below:

64. How often do you see the following (diseases, syndromes or symptoms) if you were in a "NON-HAJJ" setting?

**Non-Hajj**	Always (90% clients)	Usual (70% client)	Common (50% clients)	Sometimes (30% clients)	Rarely (10% clients)
Tonsillitis	○	○	○	○	○
Sore Throat	○	○	○	○	○
Common Cold	○	○	○	○	○
Nausea /Vomiting	○	○	○	○	○
Diarrhoea /loose stool	○	○	○	○	○
Heat Stroke	○	○	○	○	○
Heat Exhaustion	○	○	○	○	○

65. How often do you see the following (diseases, syndromes or symptoms) if you were in a "HAJJ" setting?

**In Hajj**	Always (90% clients)	Usual (70% client)	Common (50% clients)	Sometimes (30% clients)	Rarely (10% clients)
Tonsillitis	○	○	○	○	○
Sore Throat	○	○	○	○	○
Common Cold	○	○	○	○	○
Nausea /Vomiting	○	○	○	○	○
Diarrhoea /loose stool	○	○	○	○	○
Heat Stroke	○	○	○	○	○
Heat Exhaustion	○	○	○	○	○

66. What is the common causative pathogens for upper respiratory tract illnesses/infections (URTI)?
    - Viral
    - Bacterial
    - Other, please specify:

67. Would you prescribe antimicrobials for upper respiratory tract illnesses/infections (URTI)?
    - Yes, from whatever the pharmacy has
    - No, would never prescribe it for URTI
    - Yes, I will choose a specific drug
    - Other, please specify:

68. If you decide to prescribe antimicrobials for upper respiratory tract illnesses/infections (URTI), which of the following is your first choice?
    - Metronidazole
    - Flucloxacillin
    - Amoxycillin
    - Amoxycillin and Clavulanic Acid
    - Ciprofloxacin
    - Rifampicin
    - Tetracycline
    - Ethambutol
    - Isoniazid
    - Pyrazinamide
    - Colistin
    - Sulfadiazine
    - Sulfadiazine and Trimethoprim
    - Trimethoprim
    - Erythromycin
    - Clarithromycin
    - Azithromycin
    - Vancomycin
    - Cephalosporin
    - Doxycycline
    - Streptomycin
    - Sulfamethoxazole
    - Clindamycin
    - Ceftriaxone
    - Gentamicin
    - Other, please specify:

69. What is the common causative pathogens for gastrointestinal illnesses/infections (GI)?
    - Viral
    - Bacterial
    - Other, please specify:

70. Would you prescribe antimicrobials for gastrointestinal illnesses/infections (GI)?
    - Yes, from whatever the pharmacy has
    - No, would never prescribe it for GI
    - Yes, I will choose a specific drug
    - Other, please specify:

71. If you decide to prescribe antimicrobials for gastrointestinal illnesses/infections (GI), which of the following is your first choice?
    - Metronidazole
    - Flucloxacillin
    - Amoxycillin
    - Amoxycillin and Clavulanic Acid
    - Ciprofloxacin
    - Rifampicin
    - Tetracycline
    - Ethambutol
    - Isoniazid
    - Pyrazinamide
    - Colistin
    - Sulfadiazine
    - Sulfadiazine and Trimethoprim
    - Trimethoprim
    - Erythromycin
    - Clarithromycin
    - Azithromycin
    - Vancomycin
    - Cephalosporin
    - Doxycycline
    - Streptomycin
    - Sulfamethoxazole
    - Clindamycin
    - Ceftriaxone
    - Gentamicin
    - Other, please specify:

72. If you chose to Change your mind from prescribing an antimicrobial to another (in Hajj setting), What would be your reason?

	Yes	Maybe	No
The client did not like what I had prescribed	○	○	○
The client is allergic to a component of the first antimicrobial treatment	○	○	○
The pharmacist recommended that I change the antimicrobial regimen	○	○	○

If there were other reasons you would like to share please mention them below: (optional)

73. Do you know anything about the following terms?

Term	Yes, I know it well	Not sure	Never heard of it
National Institute for Health and Care Excellence – Clinical Guideline 69 (NICE CG69)	○	○	○
Centor score/criteria	○	○	○
Centres for Disease Control and Prevention: Acute Pharyngitis in Adults. Diagnosis and treatment of Group A ß-haemolytic streptococcal pharyngitis in otherwise healthy adults	○	○	○
McIsaac Decision Rule/Score	○	○	○

Are there any additional comments or notes: (optional)

74. In your opinion, should there be guidelines for prescribing antimicrobials for Upper Respiratory Tract Infections? (in general, not specific for Hajj)
    - Yes
    - No
    - Maybe

75. Why do you think so? (You may choose more than one)
    - A physician's personal experience matters
    - Health care needs to be based on evidence
    - Health services need to be standardized
    - Other, please specify:

76. **During Hajj**, should there be guidelines for prescribing antimicrobials for Upper Respiratory Tract Infection?
    - Yes
    - No
    - Maybe

77. Why do you think so? (You may choose more than one)
    - Hajj is a temporary event, no need for guidelines
    - Hajj services needs to be based on evidence
    - Presence of a guideline for Hajj saves time
    - Other, please specify:

78. If you are familiar with a guideline, have a (specific / personalised) way, or self-developed guideline for prescribing antimicrobials for upper respiratory tract infections (URTI) or gastrointestinal infections (GI) specifically for Hajj; and if you would like to share, please kindly provide details (From which: evidence, author and summery) for such guideline below: (optional)

79. If you would like to share any thoughts or Ideas to help tackle any issues related to Hajj health, prescribing / dispensing antimicrobials, or concerns; please write below: (optional)

With this, you have reached the end of our survey. Thank you for your precious time and input.

If you wish to receive further information regarding the study's result, either contact the primary investigator, details below, or provide your e-mail. If you choose to provide your e-mail, you are **consenting for the Investigators to correspond with you via E-mail as a method of electronic contact**. If so, please provide it below:

Contact information for the principal investigator via:

I. e-mail: hamid.bokhary@sydney.edu.au
II. mobile phone (call charges may apply depending on your service provider), you might need to try both numbers:
    a. Saudi Arabia: +966503619903
    b. Australia: +61414566345

Do you acknowledge that you (gave your consent/give consent) to our researchers to use the mode of electronic communication that was used to send you the link for this survey? (Your contact information will not be distributed or used except for the purpose of providing you with the electronic link for this study.)

□ I accept.

□ I do not accept.

Knowledge, Attitude and Practices of Health Care Workers for Antimicrobial Prescription and Dispense during Hajj

Dear Participant,

Thank you for the time that you'll give to fill out this form. This study helps in understanding the nature of antimicrobial prescription and dispense during the Hajj season. You are invited to participate in this survey if you are a Heath Care Worker deployed during Hajj.

This survey is part of studies conducted by researchers from The University of Sydney (Sydney, Australia) in a cooperation with their counterparts from King Abdullah Medical City (Makkah, Saudi Arabia) and The Custodian of the Two Holy Mosques Centre for Hajj and Umrah Research (Makkah, Saudi Arabia).

King Abdullah Medical City has ethically approved this survey. Further information regarding the approval is on their website. Ethics approval number 16-293.

This study **is not in any way related to your work, job requirements or assessment**. The provided information will only be used for the sole purpose of the studies and will not be distributed to other parties.

Thank you for your contribution,

Hamid Bokhary, on behalf of the Hajj Research Team.

E-mail: hamid.bokhary@sydney.edu.au

A) Declarations:

Most of this survey's questions don't require writing the answers.<br>Please just tick (✓) the appropriate box you find suitable.
☐ I understand the above statement, and will bear it in mind when answering this survey.

You will find the term "Health Care Worker" repeated in this survey.<br>Health Care Worker(s) will be defined as a person (or people) who usually work(s) or train(s) in a health care facility such as a hospital, walk-in clinic, dispensary, pathology laboratory, public health office and emergency care services. Thus would include; trainee doctors, nurses, allied HCWs, pharmacists, laboratory technicians and ambulance personnel.<br>A trainee is someone who is still undergoing training such as medical, nursing or pharmacology student undergraduates; or a postgraduate (e.g.: physician or pharmacist) in vocational specialty training.
☐ I understand the above statement, and will bear it in mind when answering this survey.

You will find the term "client" repeated in this survey.<br>Health care beneficiaries or receivers are not always patients, like those who go for routine checkups. In this survey a client is the end receiver of any health care related service; treatments, checkups, lab results, medical tests, rehabilitation, medical care, consultation and home visits are considered as such. Patients and those who come asking for medication are considered clients in this survey.<br>Hence a "Hajj client" is a "client" but presents himself as a pilgrim during Hajj season.
☐ I understand the above statement, and will bear it in mind when answering this survey.

Please bear in mind that this is ***NOT*** a test of your knowledge as an individual, but its intent is to seek the baseline knowledge of on-the-ground Health care workers in Hajj.
☐ I understand the above statement, and will bear it in mind when answering this survey.

☐ I am a Health Care Worker and I am allowed to practice my profession in Hajj.
☐ I am deployed during this Hajj season (1437H/2016G) to work as a Health Care Worker.

End of Part A

B) General Demographic Information:

B-01) Is this your first time filling this survey during this season of Hajj (1437H/2016G)?		
☐ Yes	☐ No, please mention how many times:	______ Times
If you answered "No", why are you filling it again this time?		

B-02) What is your gender?	☐ Male	☐ Female

B-03) What is your birth date? (Gregorian calendar)	Month/Year: ______ / _________G.

B-04) What is your country of birth?	

B-05) What is your nationality?	

B-06) What is your current qualification?		
☐ Still a student, including last year medical/pharmaceutical students (Internes/House Officers).		
☐ Diploma graduate.	☐ Bachelors graduate (including MBBS).	
☐ Postgraduate student.	☐ Diploma in a subspecialty. (Like Family Medicine Diploma)	
☐ Masters graduate.	☐ PhD graduate.	☐ Board qualified.
☐ Medical Specialist.	☐ Medical Consultant.	☐ Other; mention below:

B-07) What is our qualification background?		
☐ Physician (Medical Doctor).	☐ Pharmacist.	☐ Nursing.
☐ Lab scientist.	☐ Administrative (Management)	☐ Allied Health.
☐ Other; please mention:		
Year of obtaining qualification: (Gregorian Calendar)	_________G.	
Country granting the qualification:		

B-08) Are you working in the scope of your qualification?	☐ Yes
☐ Yes, but in an administrative capacity (e.g.: pharmacy manager)	☐ No, in a different scope
☐ Other, please specify:	

B-09) How many Years have you worked as a Health Care Worker in general?	Years

B-10) How many Years have you worked as a Health Care Worker in Hajj?	Years

B-11) What is the average number of days per year you work in Hajj (as a Health Care Worker in Hajj)	Days/Year

B-12) What is the average number of daily hours you put in Hajj (as a Health Care Worker in Hajj)	Hours/Day

B-13) How many clients do you see in Hajj (as a Health Care Worker in Hajj) in average?	Client/Day

B-14) Does your work involve field work (outside a health care building)?	
☐ Yes, and **it is** a mobile facility.	☐ Yes, and it is **NOT** a mobile facility.
☐ No.	

B-15) If you answered the previous question "Yes", what is the nature of your field work?	
☐ It is my main working environment	☐ I am deployed to the filed sometimes
Please specify how long your work is on the field?	Hours/Day

<u>End of Part B</u>

C) General Antimicrobial Knowledge:

C-01) Have you ever heard of **Antimicrobial Resistance**?	☐ Never heard of it
☐ Maybe or minimal information	☐ Yes, I know it well
☐ Other, please mention:	
If you answered “Yes” or “Maybe”, where did you hear it from? (you may choose more than one)	
☐ My degree course	☐ Newspaper
☐ Newsletter	☐ Web
☐ Other, please specify:	

C-02) Have you ever heard of **Antimicrobial Stewardship Programs**	☐ Never heard of it
☐ Maybe or minimal information	☐ Yes, I know it well
☐ Other, please mention:	
If you answered “Yes” or “Maybe”, where did you hear it from? (you may choose more than one)	
☐ My degree course	☐ Newspaper
☐ Newsletter	☐ Web
☐ Other, please specify:	

C-03) What organisms do you think antimicrobials help treat (in general, not specific to Hajj)? (you may choose more than one)			
Organism	Yes	No	Maybe
Viruses	☐	☐	☐
Bacteria	☐	☐	☐
Fungi	☐	☐	☐
Parasites	☐	☐	☐
Insects	☐	☐	☐

C-04) Do you think antimicrobials should be available to the public **without** restrictions?	
☐ No, it should be restricted	☐ Yes, no restrictions should apply.

C-05) If you answered “No” above, what restrictions should be applied for dispensing antimicrobials? (You may choose more than one)			
Restriction	Yes	Maybe	No
Antimicrobials need a doctor’s opinion for consideration.	☐	☐	☐
Dispensaries should only give antimicrobials if presented with a prescription.	☐	☐	☐
Only one type of antimicrobials should be dispensed to a recipient.	☐	☐	☐
Manufacturing of antimicrobials should be regulated and supervised.	☐	☐	☐
Regular and unplanned inspections to Pharmacies.	☐	☐	☐
Doctors should only prescribe antimicrobials if an evidence based criterion was achieved.	☐	☐	☐
Other(s), please mention:			

C-06) Sometimes antimicrobials would “not to work” anymore against the same illness, what would the reason for that be? (you may choose more than one)			
Cause	Yes	Maybe	No
The **human body** builds up immunity/resistance against the antimicrobial	☐	☐	☐
The **organisms** build up immunity/resistance against the antimicrobial	☐	☐	☐
The **antimicrobial** efficacy decreases against the illness over time	☐	☐	☐
Other(s), please mention:			

C-07) what best describes your authority in regards to antimicrobials?
☐ I **can only** prescribe antimicrobials. (Please go to Page 8)
☐ I **can only** dispense antimicrobials. (Please got to Page 11)
☐ I **can do both**, prescribe and dispense antimicrobials. (Please go to the next page)
☐ I **cannot** prescribe antimicrobials and I **cannot** dispense antimicrobials. (Thank you for your time and input, you can now stop and give the survey back to the researchers)

<u>End of Part C</u>

D) Knowledge, Attitude and Practices; Antimicrobial Dispensing Respondents:

D-01) How many medicines do you dispense every day?					
☐ 0-99	☐ 100-199	☐ 200-299	☐ 300-399	☐ 400-499	☐ >499

D-02) How many antimicrobials do you dispense every day?					
☐ 0-49	☐ 50-99	☐ 100-149	☐ 150-199	☐ 200-249	☐ >249

D-03) What are the common antimicrobials you recommend to your clients? (Fill in with numbers)<br>Antimicrobial	1 to 17 (1 is the commonest and 17 least common)	Is it a broad-spectrum antimicrobial?
Amoxicillin		□ Yes/□ No/□ Maybe
Amoxicillin/clavulanic acid		□ Yes/□ No/□ Maybe
Ampicillin		□ Yes/□ No/□ Maybe
Azithromycin		□ Yes/□ No/□ Maybe
Cefuroxime		□ Yes/□ No/□ Maybe
Cefexitin		□ Yes/□ No/□ Maybe
Cephalexin		□ Yes/□ No/□ Maybe
Clarithromycin		□ Yes/□ No/□ Maybe
Ciprofloxacin		□ Yes/□ No/□ Maybe
Dicloxacillin		□ Yes/□ No/□ Maybe
Doxycyclin		□ Yes/□ No/□ Maybe
Erythromycin		□ Yes/□ No/□ Maybe
Flucloxacillin		□ Yes/□ No/□ Maybe
Norfloxacillin		□ Yes/□ No/□ Maybe
Ofloxacillin		□ Yes/□ No/□ Maybe
Roxithromycin		□ Yes/□ No/□ Maybe
Clindamycin		□ Yes/□ No/□ Maybe

D-04) What are the common conditions you dispense antimicrobials without a prescription for? Please tick (✓) the appropriate box		List the antimicrobials(s) you dispense over the counter for this condition
Cold and Flu	□	
Sore throat	□	
Wet Cough	□	
Dry cough	□	
Runny nose	□	
Skin/Foot infection	□	
Urinary tract infection	□	
Diarrhoea	□	
Dysentery	□	
Toothache	□	
Earache	□	
Fever	□	
Eye infection	□	
Other (please specify)		

D-05) Please answer the following questions			
Do, Does or Will …	Yes	No	Maybe
Antimicrobials treat (or speed up the recovery from) most coughs, colds and flu?	☐	☐	☐
Bacteria causes upper respiratory tract infections more than viruses	☐	☐	☐
Human can be resistant to antimicrobials.	☐	☐	☐
Antimicrobials are effective against viruses	☐	☐	☐
The antimicrobial use in animals can reduce their effectiveness in humans?	☐	☐	☐
Inappropriate use of antimicrobials contributes to development of resistance	☐	☐	☐
Antimicrobials can cause secondary infections after killing good bacteria present in our system	☐	☐	☐
Misuse of antimicrobials can lead to a loss of sensitivity of an antimicrobial to a specific pathogen	☐	☐	☐
Use of antimicrobials over the counter without prescription contribute to development of resistance	☐	☐	☐
Common infections and minor injuries which have been treatable for decades can once again kill the clients, due to antimicrobial resistance.	☐	☐	☐
You recommend the clients to stop taking antimicrobials when they start feeling better	☐	☐	☐
You have weekly/monthly sales target to achieve	☐	☐	☐
By not dispensing antimicrobials over the counter will negatively affect sales targets	☐	☐	☐
Clients buy antimicrobials over the counter from another pharmacy if you don't dispense on their demand	☐	☐	☐
You dispense antimicrobials over the counter because your clients can't afford doctor's fee	☐	☐	☐
Dispensing antimicrobials over the counter without prescription is legal practice in Saudi Arabia	☐	☐	☐
When dispensing antimicrobials, I check clients's drug allergy status	☐	☐	☐
Antimicrobials do not have side effects	☐	☐	☐
You dispense over the counter antimicrobials because the clients expects it	☐	☐	☐
You think most of the upper respiratory tract infection will be self-cured even without use of antimicrobials	☐	☐	☐

D-06) What are the common microorganisms which cause different infections given below						
Infection site	Upper respiratory tract infections	Lower respiratory tract infections	Diarrhoea	Dysentery	Skin / Foot infection	Urinary Tract Infection (UTI)
Staphylococcus aureus						
Streptococcus pneumoniae						

Infection site	Upper respiratory tract infections	Lower respiratory tract infections	Diarrhoea	Dysentery	Skin / Foot infection	Urinary Tract Infection (UTI)
Neisseria meningitids						
Haemophilus influenzae						
Pseudomonas aurogenosa						
Escherchia Coli						
Klebsiella pneumoniae						
Methicillin Resistant Staphylococcus aureus (MRSA)						
Legionella spp.						
Chlamydia spp.						
Moraxella catarrhalis						
Salmonella spp.						
Mycobacterium avium complex						
Mycobacterium tuberculosis						
Mycoplasma						
Enterococcus spp.						

End of Part D

E) Background Information for Antimicrobial Prescribing Respondents:

E-01) Are you a physician Health Care Worker?	☐ Yes.
☐ No. Please specify	

E-02) What is your speciality? (Please choose **only one**)	
☐ Intern, Year 7 Medical School	☐ General Practitioner (GP), Medical school graduate who's not enrolled in a speciality yet
☐ Family Medicine	
☐ ENT (Ear, Nose and Throat)	☐ Public Health/Community Medicine
☐ Emergency Medicine	☐ Paediatrics
☐ Surgery	☐ Internal Medicine
☐ Other, please specify	

E-03) Do antimicrobials treat **Viruses** (in Hajj setting)?			
☐ Yes	☐ No	☐ Maybe	
If you answered "No" or "Maybe" would you prescribe anyway?		☐ No	☐ Yes, all the time
☐ Yes, in specific situations. Mention please:			
Regardless of your answer, why would you prescribe (or not) antimicrobials? (You may choose more than one)	☐ It is the main choice of treatment.	☐ To stop further deterioration of the client's clinical situation	
☐ There is **no** evidence supporting GIVING antimicrobials during Hajj against **Viruses**	☐ There is **no** evidence supporting NOT GIVING antimicrobials during Hajj against **Viruses**		
☐ Other, please mention:			

E-04) Do antimicrobials treat **Bacteria** (in Hajj setting)?			
☐ Yes	☐ No	☐ Maybe	
If you answered "No" or "Maybe" would you prescribe anyway?		☐ No	☐ Yes, all the time
☐ Yes, in specific situations. Mention please:			
Regardless of your answer, why would you prescribe (or not) antimicrobials? (You may choose more than one)	☐ It is the main choice of treatment.	☐ To stop further deterioration of the client's clinical situation	
☐ There is **no** evidence supporting GIVING antimicrobials during Hajj against **Bacteria**	☐ There is **no** evidence supporting NOT GIVING antimicrobials during Hajj against **Bacteria**		
☐ Other, please mention:			

E-05) Do antimicrobials treat **Fungi** (in Hajj setting)?			
☐ Yes	☐ No	☐ Maybe	
If you answered "No" or "Maybe" would you prescribe anyway?		☐ No	☐ Yes, all the time
☐ Yes, in specific situations. Mention please:			
Regardless of your answer, why would you prescribe (or not) antimicrobials? (You may choose more than one)	☐ It is the main choice of treatment.	☐ To stop further deterioration of the client's clinical situation	
☐ There is **no** evidence supporting GIVING antimicrobials during Hajj against **Fungi**	☐ There is **no** evidence supporting NOT GIVING antimicrobials during Hajj against **Fungi**		
☐ Other, please mention:			

E-06) Do antimicrobials treat **Parasites** (in Hajj setting)?			
☐ Yes	☐ No	☐ Maybe	
If you answered "No" or "Maybe" would you prescribe anyway?		☐ No	☐ Yes, all the time
☐ Yes, in specific situations. Mention please:			
Regardless of your answer, why would you prescribe (or not) antimicrobials? (You may choose more than one)	☐ It is the main choice of treatment.	☐ To stop further deterioration of the client's clinical situation	
☐ There is **no** evidence supporting GIVING antimicrobials during Hajj against **Parasites**	☐ There is **no** evidence supporting NOT GIVING antimicrobials during Hajj against **Parasites**		
☐ Other, please mention:			

E-07) Do antimicrobials treat **Insects** (in Hajj setting)?			
☐ Yes	☐ No	☐ Maybe	
If you answered "No" or "Maybe" would you prescribe anyway?		☐ No	☐ Yes, all the time
☐ Yes, in specific situations. Mention please:			
Regardless of your answer, why would you prescribe (or not) antimicrobials? (You may choose more than one)	☐ It is the main choice of treatment.	☐ To stop further deterioration of the client's clinical situation	
☐ There is **no** evidence supporting GIVING antimicrobials during Hajj against **Insects**	☐ There is **no** evidence supporting NOT GIVING antimicrobials during Hajj against **Insects**		
☐ Other, please mention:			

E-08) Are you crowded with clients during Hajj?	
☐ No, it is like any other given day.	☐ Yes, but it is bearable and manageable.
☐ Yes, in an intolerable amount	☐ Other, please mention below
Regardless of your answer, does your situation above affect your decision on prescribing antimicrobials?	
☐ No, it does not.	☐ Yes, I tend to over/under prescribe antimicrobials during Hajj.
☐ Other, please mention:	

E-09) Have you heard reports of Antimicrobial Resistance for the antimicrobials below? (You may answer more than one)

Antimicrobial	Yes	No	Maybe	Other / Comments
Penicillin	☐	☐	☐	
Metronidazole	☐	☐	☐	
Flucloxacillin	☐	☐	☐	
Amoxycillin	☐	☐	☐	
Amoxycillin with clavulanic acid	☐	☐	☐	
Ciprofloxacin	☐	☐	☐	
Rifampicin	☐	☐	☐	
Tetracyclines	☐	☐	☐	
Doxycycline	☐	☐	☐	
Ethambutol	☐	☐	☐	
Isoniazid	☐	☐	☐	
Pyrazinamide	☐	☐	☐	
Streptomycin	☐	☐	☐	
Colistin	☐	☐	☐	
Sulfadiazine	☐	☐	☐	
Trimethoprim	☐	☐	☐	
Trimethoprim with Sulfamethoxazole	☐	☐	☐	
Erythromycin	☐	☐	☐	
Clarithromycin	☐	☐	☐	
Azithromycin	☐	☐	☐	
Clindamycin	☐	☐	☐	
Vancomycin	☐	☐	☐	
Cephalosporins	☐	☐	☐	
Gentamicin	☐	☐	☐	

E-09) Do you treat clients for Upper Respiratory Tract Infections?	
☐ No, it is **NOT** within the scope of my speciality	☐ Yes, it is within the scope of my speciality
☐ Yes, but it is **NOT** within the scope of my speciality	
If You answered “Yes”, please continue to the next part.	

End of Part E

F) Knowledge, Attitude and Practices; Antimicrobial Prescription during Hajj for Upper Respiratory Tract infections:

F-01) Would you prescribe antimicrobials to all of your Hajj clients (without regards to their situation) when treating them from Upper Respiratory Tract Infections?	
☐ Yes	☐ No
Regardless of your answer, why?	☐ I have to assess each client as a unique case, as long as it takes to do so, bearing in mind Hajj settings are usually crowded
☐ I **DO NOT** have time to assess each client's detailed case, because Hajj settings are usually crowded	☐ Other, please mention below.

F-02) After prescribing an antimicrobial for Upper Respiratory Tract Infections to a **Hajj client**, have you ever considered advising them to delay taking it (not starting regimen immediately)?	
☐ Yes, always	☐ No, never.
☐ Yes, for some clients. Please elaborate: the percentage from total Upper Respiratory Tract Infections antimicrobial prescriptions?	_____ %
Regardless of your answer, why? (You may choose more than one answer)	☐ If I wanted to delay treatment, I would not have prescribed antimicrobials in the first place
☐ I want to give the client's natural immunity a chance to fight the illness	☐ Other, please mention below.

F-03) From the following practices, what would you offer your clients during Hajj if you intend not to prescribe antimicrobials immediately? (You may choose more than one)				
Practice	When refusing to prescribe antimicrobials	When delaying prescribing antimicrobials	When giving antimicrobials immediately	Never do it
Reassurance that antimicrobials are not needed immediately because they are likely to make little difference to symptoms and may have side effects, for example, diarrhoea, vomiting and rash	☐	☐	☐	☐
Advice to re-consult if there is a significant worsening or prolongation of symptoms	☐	☐	☐	☐

Advice about the usual natural history of the illness, including the average total length of the illness (before and after seeing you)	☐	☐	☐	☐
Advice about managing symptoms, including fever (particularly analgesics and antipyretics)	☐	☐	☐	☐

F-04) Would you prescribe antimicrobials to the following clinical scenarios during Hajj?						
Scenario	Yes, if it (was/with other scenarios):					Never
	Alone	1 other	2 others	3 or more	absent	
Old / geriatric clients (>55 years old)	☐	☐	☐	☐	☐	☐
Young clients (2 to <15 years old)	☐	☐	☐	☐	☐	☐
Very Young clients (<2 years old)	☐	☐	☐	☐	☐	☐
client is systematically unwell	☐	☐	☐	☐	☐	☐
If clinical assessment suggests serious illness/complications	☐	☐	☐	☐	☐	☐
If client has a pre-existing comorbidity (heart, lung, renal, liver …etc.)	☐	☐	☐	☐	☐	☐
If client has a pre-existing chronic medical illness (diabetes …etc.)	☐	☐	☐	☐	☐	☐
History of hospitalization in previous year	☐	☐	☐	☐	☐	☐
Bilateral acute otitis media in children younger than 2 years	☐	☐	☐	☐	☐	☐
Acute otitis media in children with otorrhoea	☐	☐	☐	☐	☐	☐
Three or more Centor score/criteria are present	☐	☐	☐	☐	☐	☐
Fever (>38.0 C^{o})	☐	☐	☐	☐	☐	☐
The presence of exudative tonsillitis (Pus on tonsils)	☐	☐	☐	☐	☐	☐
The presence of lymphadenopathy or lymph node enlargement in relevant areas	☐	☐	☐	☐	☐	☐
Cough	☐	☐	☐	☐	☐	☐
Use of oral glucocorticoids	☐	☐	☐	☐	☐	☐
Other, please mention below. Please tick (✓) appropriately for each written scenario						
	☐	☐	☐	☐	☐	☐
	☐	☐	☐	☐	☐	☐
	☐	☐	☐	☐	☐	☐
	☐	☐	☐	☐	☐	☐

F-05) Is Tonsillitis considered to be an Upper Respiratory Tract Infection?			
☐ Yes	☐ No	☐ Maybe	☐ I don’t know
Regardless of the answer: how many cases (overall) do you see during Hajj season			_____clients/Day
What is the average total length of the illness, including the time before the client came to see you			_______ Days
What is the common causative pathogens?		☐ Viral	☐ Bacterial
☐ Other, please mention:			
If you decide to prescribe antimicrobials for this medical condition, which of the following is your first choice? (choose more than one for combinations)			☐ Penicillin
☐ Metronidazole	☐ Flucloxacillin	☐ Amoxycillin	☐ Clavulanic Acid
☐ Ciprofloxacin	☐ Rifampicin	☐ Tetracycline	☐ Doxycycline
☐ Ethambutol	☐ Isoniazid	☐ Pyrazinamide	☐ Streptomycin
☐ Colistin	☐ Sulfadiazine	☐ Trimethoprim	☐ Sulfamethoxazole
☐ Erythromycin	☐ Clarithromycin	☐ Azithromycin	☐ Clindamycin
☐ Vancomycin	☐ Cephalosporins	☐ Gentamicin	☐ Ceftriaxone
☐ Whatever is available in the pharmacy	☐ would never prescribe antimicrobials for this condition	☐ Other, please mention:	

F-06) Is Sinusitis considered to be an Upper Respiratory Tract Infection?			
☐ Yes	☐ No	☐ Maybe	☐ I don’t know
Regardless of the answer: how many cases (overall) do you see during Hajj season			_____clients/Day
What is the average total length of the illness, including the time before the client came to see you			_______ Days
What is the common causative pathogens?		☐ Viral	☐ Bacterial
☐ Other, please mention:			
If you decide to prescribe antimicrobials for this medical condition, which of the following is your first choice? (choose more than one for combinations)			☐ Penicillin
☐ Metronidazole	☐ Flucloxacillin	☐ Amoxycillin	☐ Clavulanic Acid
☐ Ciprofloxacin	☐ Rifampicin	☐ Tetracycline	☐ Doxycycline
☐ Ethambutol	☐ Isoniazid	☐ Pyrazinamide	☐ Streptomycin
☐ Colistin	☐ Sulfadiazine	☐ Trimethoprim	☐ Sulfamethoxazole
☐ Erythromycin	☐ Clarithromycin	☐ Azithromycin	☐ Clindamycin
☐ Vancomycin	☐ Cephalosporins	☐ Gentamicin	☐ Ceftriaxone
☐ Whatever is available in the pharmacy	☐ would never prescribe antimicrobials for this condition	☐ Other, please mention:	

F-07) Is Rhinitis considered to be an Upper Respiratory Tract Infection?			
☐ Yes	☐ No	☐ Maybe	☐ I don't know
Regardless of the answer: how many cases (overall) do you see during Hajj season			_____clients/Day
What is the average total length of the illness, including the time before the client came to see you			_______ Days
What is the common causative pathogens?		☐ Viral	☐ Bacterial
☐ Other, please mention:			
If you decide to prescribe antimicrobials for this medical condition, which of the following is your first choice? (choose more than one for combinations)			☐ Penicillin
☐ Metronidazole	☐ Flucloxacillin	☐ Amoxycillin	☐ Clavulanic Acid
☐ Ciprofloxacin	☐ Rifampicin	☐ Tetracycline	☐ Doxycycline
☐ Ethambutol	☐ Isoniazid	☐ Pyrazinamide	☐ Streptomycin
☐ Colistin	☐ Sulfadiazine	☐ Trimethoprim	☐ Sulfamethoxazole
☐ Erythromycin	☐ Clarithromycin	☐ Azithromycin	☐ Clindamycin
☐ Vancomycin	☐ Cephalosporins	☐ Gentamicin	☐ Ceftriaxone
☐ Whatever is available in the pharmacy	☐ would never prescribe antimicrobials for this condition	☐ Other, please mention:	

F-08) Is Pharyngitis considered to be an Upper Respiratory Tract Infection?			
☐ Yes	☐ No	☐ Maybe	☐ I don't know
Regardless of the answer: how many cases (overall) do you see during Hajj season			_____clients/Day
What is the average total length of the illness, including the time before the client came to see you			_______ Days
What is the common causative pathogens?		☐ Viral	☐ Bacterial
☐ Other, please mention:			
If you decide to prescribe antimicrobials for this medical condition, which of the following is your first choice? (choose more than one for combinations)			☐ Penicillin
☐ Metronidazole	☐ Flucloxacillin	☐ Amoxycillin	☐ Clavulanic Acid
☐ Ciprofloxacin	☐ Rifampicin	☐ Tetracycline	☐ Doxycycline
☐ Ethambutol	☐ Isoniazid	☐ Pyrazinamide	☐ Streptomycin
☐ Colistin	☐ Sulfadiazine	☐ Trimethoprim	☐ Sulfamethoxazole
☐ Erythromycin	☐ Clarithromycin	☐ Azithromycin	☐ Clindamycin
☐ Vancomycin	☐ Cephalosporins	☐ Gentamicin	☐ Ceftriaxone
☐ Whatever is available in the pharmacy	☐ would never prescribe antimicrobials for this condition	☐ Other, please mention:	

F-09) Is Otitis media considered to be an Upper Respiratory Tract Infection?			
☐ Yes	☐ No	☐ Maybe	☐ I don't know
Regardless of the answer: how many cases (overall) do you see during Hajj season			_____clients/Day
What is the average total length of the illness, including the time before the client came to see you			_______ Days
What is the common causative pathogens?		☐ Viral	☐ Bacterial
☐ Other, please mention:			
If you decide to prescribe antimicrobials for this medical condition, which of the following is your first choice? (choose more than one for combinations)			☐ Penicillin
☐ Metronidazole	☐ Flucloxacillin	☐ Amoxycillin	☐ Clavulanic Acid
☐ Ciprofloxacin	☐ Rifampicin	☐ Tetracycline	☐ Doxycycline
☐ Ethambutol	☐ Isoniazid	☐ Pyrazinamide	☐ Streptomycin
☐ Colistin	☐ Sulfadiazine	☐ Trimethoprim	☐ Sulfamethoxazole
☐ Erythromycin	☐ Clarithromycin	☐ Azithromycin	☐ Clindamycin
☐ Vancomycin	☐ Cephalosporins	☐ Gentamicin	☐ Ceftriaxone
☐ Whatever is available in the pharmacy	☐ would never prescribe antimicrobials for this condition	☐ Other, please mention:	

F-10) Is Otitis interna considered to be an Upper Respiratory Tract Infection?			
☐ Yes	☐ No	☐ Maybe	☐ I don't know
Regardless of the answer: how many cases (overall) do you see during Hajj season			_____clients/Day
What is the average total length of the illness, including the time before the client came to see you			_______ Days
What is the common causative pathogens?		☐ Viral	☐ Bacterial
☐ Other, please mention:			
If you decide to prescribe antimicrobials for this medical condition, which of the following is your first choice? (choose more than one for combinations)			☐ Penicillin
☐ Metronidazole	☐ Flucloxacillin	☐ Amoxycillin	☐ Clavulanic Acid
☐ Ciprofloxacin	☐ Rifampicin	☐ Tetracycline	☐ Doxycycline
☐ Ethambutol	☐ Isoniazid	☐ Pyrazinamide	☐ Streptomycin
☐ Colistin	☐ Sulfadiazine	☐ Trimethoprim	☐ Sulfamethoxazole
☐ Erythromycin	☐ Clarithromycin	☐ Azithromycin	☐ Clindamycin
☐ Vancomycin	☐ Cephalosporins	☐ Gentamicin	☐ Ceftriaxone
☐ Whatever is available in the pharmacy	☐ would never prescribe antimicrobials for this condition	☐ Other, please mention:	

F-11) Is Otitis externa considered to be an Upper Respiratory Tract Infection?			
☐ Yes	☐ No	☐ Maybe	☐ I don't know
Regardless of the answer: how many cases (overall) do you see during Hajj season			_____clients/Day
What is the average total length of the illness, including the time before the client came to see you			________ Days
What is the common causative pathogens?		☐ Viral	☐ Bacterial
☐ Other, please mention:			
If you decide to prescribe antimicrobials for this medical condition, which of the following is your first choice? (choose more than one for combinations)			☐ Penicillin
☐ Metronidazole	☐ Flucloxacillin	☐ Amoxycillin	☐ Clavulanic Acid
☐ Ciprofloxacin	☐ Rifampicin	☐ Tetracycline	☐ Doxycycline
☐ Ethambutol	☐ Isoniazid	☐ Pyrazinamide	☐ Streptomycin
☐ Colistin	☐ Sulfadiazine	☐ Trimethoprim	☐ Sulfamethoxazole
☐ Erythromycin	☐ Clarithromycin	☐ Azithromycin	☐ Clindamycin
☐ Vancomycin	☐ Cephalosporins	☐ Gentamicin	☐ Ceftriaxone
☐ Whatever is available in the pharmacy	☐ would never prescribe antimicrobials for this condition	☐ Other, please mention:	

F-12) Is Laryngitis considered to be an Upper Respiratory Tract Infection?			
☐ Yes	☐ No	☐ Maybe	☐ I don't know
Regardless of the answer: how many cases (overall) do you see during Hajj season			_____clients/Day
What is the average total length of the illness, including the time before the client came to see you			________ Days
What is the common causative pathogens?		☐ Viral	☐ Bacterial
☐ Other, please mention:			
If you decide to prescribe antimicrobials for this medical condition, which of the following is your first choice? (choose more than one for combinations)			☐ Penicillin
☐ Metronidazole	☐ Flucloxacillin	☐ Amoxycillin	☐ Clavulanic Acid
☐ Ciprofloxacin	☐ Rifampicin	☐ Tetracycline	☐ Doxycycline
☐ Ethambutol	☐ Isoniazid	☐ Pyrazinamide	☐ Streptomycin
☐ Colistin	☐ Sulfadiazine	☐ Trimethoprim	☐ Sulfamethoxazole
☐ Erythromycin	☐ Clarithromycin	☐ Azithromycin	☐ Clindamycin
☐ Vancomycin	☐ Cephalosporins	☐ Gentamicin	☐ Ceftriaxone
☐ Whatever is available in the pharmacy	☐ would never prescribe antimicrobials for this condition	☐ Other, please mention:	

F-13) Is Esophagitis considered to be an Upper Respiratory Tract Infection?			
☐ Yes	☐ No	☐ Maybe	☐ I don't know
Regardless of the answer: how many cases (overall) do you see during Hajj season			_____clients/Day
What is the average total length of the illness, including the time before the client came to see you			_______ Days
What is the common causative pathogens?		☐ Viral	☐ Bacterial
☐ Other, please mention:			
If you decide to prescribe antimicrobials for this medical condition, which of the following is your first choice? (choose more than one for combinations)			☐ Penicillin
☐ Metronidazole	☐ Flucloxacillin	☐ Amoxycillin	☐ Clavulanic Acid
☐ Ciprofloxacin	☐ Rifampicin	☐ Tetracycline	☐ Doxycycline
☐ Ethambutol	☐ Isoniazid	☐ Pyrazinamide	☐ Streptomycin
☐ Colistin	☐ Sulfadiazine	☐ Trimethoprim	☐ Sulfamethoxazole
☐ Erythromycin	☐ Clarithromycin	☐ Azithromycin	☐ Clindamycin
☐ Vancomycin	☐ Cephalosporins	☐ Gentamicin	☐ Ceftriaxone
☐ Whatever is available in the pharmacy	☐ would never prescribe antimicrobials for this condition	☐ Other, please mention:	

F-14) Is Common cold considered to be an Upper Respiratory Tract Infection?			
☐ Yes	☐ No	☐ Maybe	☐ I don't know
Regardless of the answer: how many cases (overall) do you see during Hajj season			_____clients/Day
What is the average total length of the illness, including the time before the client came to see you			_______ Days
What is the common causative pathogens?		☐ Viral	☐ Bacterial
☐ Other, please mention:			
If you decide to prescribe antimicrobials for this medical condition, which of the following is your first choice? (choose more than one for combinations)			☐ Penicillin
☐ Metronidazole	☐ Flucloxacillin	☐ Amoxycillin	☐ Clavulanic Acid
☐ Ciprofloxacin	☐ Rifampicin	☐ Tetracycline	☐ Doxycycline
☐ Ethambutol	☐ Isoniazid	☐ Pyrazinamide	☐ Streptomycin
☐ Colistin	☐ Sulfadiazine	☐ Trimethoprim	☐ Sulfamethoxazole
☐ Erythromycin	☐ Clarithromycin	☐ Azithromycin	☐ Clindamycin
☐ Vancomycin	☐ Cephalosporins	☐ Gentamicin	☐ Ceftriaxone
☐ Whatever is available in the pharmacy	☐ would never prescribe antimicrobials for this condition	☐ Other, please mention:	

F-15) Is Bronchiolitis considered to be an Upper Respiratory Tract Infection?			
☐ Yes	☐ No	☐ Maybe	☐ I don't know
Regardless of the answer: how many cases (overall) do you see during Hajj season			_____clients/Day
What is the average total length of the illness, including the time before the client came to see you			_______ Days
What is the common causative pathogens?		☐ Viral	☐ Bacterial
☐ Other, please mention:			
If you decide to prescribe antimicrobials for this medical condition, which of the following is your first choice? (choose more than one for combinations)			☐ Penicillin
☐ Metronidazole	☐ Flucloxacillin	☐ Amoxycillin	☐ Clavulanic Acid
☐ Ciprofloxacin	☐ Rifampicin	☐ Tetracycline	☐ Doxycycline
☐ Ethambutol	☐ Isoniazid	☐ Pyrazinamide	☐ Streptomycin
☐ Colistin	☐ Sulfadiazine	☐ Trimethoprim	☐ Sulfamethoxazole
☐ Erythromycin	☐ Clarithromycin	☐ Azithromycin	☐ Clindamycin
☐ Vancomycin	☐ Cephalosporins	☐ Gentamicin	☐ Ceftriaxone
☐ Whatever is available in the pharmacy	☐ would never prescribe antimicrobials for this condition	☐ Other, please mention:	

F-16) Is Bronchitis considered to be an Upper Respiratory Tract Infection?			
☐ Yes	☐ No	☐ Maybe	☐ I don't know
Regardless of the answer: how many cases (overall) do you see during Hajj season			_____clients/Day
What is the average total length of the illness, including the time before the client came to see you			_______ Days
What is the common causative pathogens?		☐ Viral	☐ Bacterial
☐ Other, please mention:			
If you decide to prescribe antimicrobials for this medical condition, which of the following is your first choice? (choose more than one for combinations)			☐ Penicillin
☐ Metronidazole	☐ Flucloxacillin	☐ Amoxycillin	☐ Clavulanic Acid
☐ Ciprofloxacin	☐ Rifampicin	☐ Tetracycline	☐ Doxycycline
☐ Ethambutol	☐ Isoniazid	☐ Pyrazinamide	☐ Streptomycin
☐ Colistin	☐ Sulfadiazine	☐ Trimethoprim	☐ Sulfamethoxazole
☐ Erythromycin	☐ Clarithromycin	☐ Azithromycin	☐ Clindamycin
☐ Vancomycin	☐ Cephalosporins	☐ Gentamicin	☐ Ceftriaxone
☐ Whatever is available in the pharmacy	☐ would never prescribe antimicrobials for this condition	☐ Other, please mention:	

F-17) If you chose to change your mind about prescribing an antimicrobial other than your first choice, what would be the reason for that? (You may choose more than one)			
Reason	Yes	No	Maybe
The client did not like what I had prescribed	◻	◻	◻
The client is allergic to a component of the first antimicrobial treatment	◻	◻	◻
The pharmacist recommended that I change the antimicrobial regimen	◻	◻	◻
Other, please specify:			

F-18) Do you know anything about the following terms?				
Term	Yes I know it well	Maybe or minimal information	Never heard of it	Other answer or comment
The Australian guideline for prevention, diagnosis and management of acute rheumatic fever and rheumatic heart disease	☐	☐	☐	
National Institute for Health and Care Excellence - Clinical Guideline 69 (NICE CG69)	☐	☐	☐	
Infectious Diseases Society of America Guideline for the Diagnosis and Management of Group A Streptococcal Pharyngitis	☐	☐	☐	
University of Michigan Health System Guidelines: Pharyngitis	☐	☐	☐	
Guidelines for prevention and control of group A streptococcal infection in acute healthcare and maternity settings in the UK	☐	☐	☐	
Centor score/criteria	☐	☐	☐	
Institute for Clinical Systems Improvement Health Care Guideline: Diagnosis and Treatment of Respiratory Illness in Children and Adults	☐	☐	☐	
Centres for Disease Control and Prevention: Acute Pharyngitis in Adults. Diagnosis and treatment of Group A ß-haemolytic streptococcal pharyngitis in otherwise healthy adults	☐	☐	☐	
The Interdisciplinary Section for Antibiotic Resistance Control (ISKRA) guidelines on sore throat – Croatian national guidelines	☐	☐	☐	

F-19) Should there be guidelines for prescribing antimicrobials for **Upper Respiratory Tract Infection?** (in general not specific for Hajj)	
☐ Yes	☐ No
Why? (you may choose more than one)	☐ A physician personal experience matters.
☐ Health care needs to be based on evidence	☐ Health services need to be standardized
☐ Other, please mention:	

F-20) **During Hajj**, should there be guidelines for prescribing antimicrobials for Upper Respiratory Tract Infection?	
☐ Yes	☐ No
Why? (you may choose more than one)	☐ Hajj is a temporary, no need for guidelines.
☐ Hajj services needs to be based on evidence	☐ Presence of a guideline in Hajj saves time.
☐ Other, please mention:	

F-21) If you are familiar with a guideline for prescribing antimicrobials for URTI specifically for Hajj, please provide details for such guideline. (From which: evidence, author and summery)

F-21) If you have a (specific/personalised) way or self-developed guideline for prescribing antimicrobials for Upper Respiratory Tract Infections and you would like to share, please write below.

End of Part E

With this, you have reached the end of our survey.<br>Thank you for your precious time and input.<br>Please hand the survey sheet back to the researchers.

☐ I wish to receive further information regarding the study results, please do so through the contact information below. (this is optional for your own interest)
